# Vaccination with endosomal unknown epitopes produces therapeutic response in rheumatoid arthritis patients and modulates adjuvant arthritis of rats

**DOI:** 10.1186/s12967-016-0908-7

**Published:** 2016-06-07

**Authors:** Innocenzo Caruso, Salvatore Santandrea, Mariarita Gismondo, Alessandra Lombardi, Franco Montrone, Enzo Massimo Caruso, Piercarlo Sarzi Puttini

**Affiliations:** Past Chief Rheumatology Unit, L. Sacco Hospital Milano Italy, Corso Italia 11, 20122 Milan, Italy; Rheumatology UOC, L. Sacco Hospital Milano Italy, Milan, Italy; Director of microbiology chair, University of Milan, L. Sacco Hospital Milano Italy, Milan, Italy; Microbiology and Virology UO, L. Sacco Hospital milano Italy, Milan, Italy; Associazione Italiana Contro l’Artrite (AICA), Milan, Italy; Orthopedic Unit, Istituto Clinico San Siro, Milan, Italy; Chief of rheumatology Unit. L. Sacco Hospital Milano Italy, Milan, Italy

**Keywords:** Ultrafiltrate from PBMCs, Autologous immunotherapy, Arthritogenic epitope, Immunotherapeutic vaccine, Bystander antigen, Bystander immunotherapy

## Abstract

**Background:**

Our previous results showed that intrasynovial Rifamycin SV caused the lysis of synoviocites and freed the autoantigens which in turn stimulated the immunoregulatory rather than autoreactive T cell response in rheumatoid patients. Here, we hypothesize that disruption in vitro of peripheral blood mononuclear cells, by freeze/thawing or by lytic action of Rifamycin SV, would induce the release of endosomal pathogenic autoantigens from APCs present in the circulation, which could then be isolated from degrading enzymes by ultrafiltration.

**Methods:**

The preparation of the ultrafiltrates are based on the rupture of PBMCs (5 × 10^6^ cells/mL) by the addition of Rifamycin SV in culture (250 μg/mL), which causes the lysis of 90 % of the cells in 3 h, or by three cycles of freeze/thawing of the PBMC, from −80 °C to room temperature. The lysate and the fragmented cells were then centrifuged and ultrafiltered by passage through a filtration device with a cut-off of 10 kDa. Also the synovial fluid was subjected to ultrafiltration.

**Results and conclusions:**

At clinical monitoring of the 30th day, 22/58 (38 %) patients subcutaneously treated with the autologous ultrafiltrate prepared by the freeze/thawing of PBMCs reached an ACR20. Comparable results were obtained with the other two ultrafiltrates.

*Cell cultures* The addition of ultrafiltrates to rheumatoid PBMCs cultures led to the upregulation of a marker for T-regulatory cells, and downregulation of a cell proliferation marker; changes that together have the meaning of a global immunomodulatory response and that only a specific antigen (ultrafiltrate UF-f/t) might induce in the rheumatoid patient, probably by activating pre-existing protective network.

*Experimental arthritis* All the ultrafiltrates except that prepared by Rifamycin SV were able to modulate the adjuvant arthritis in rats. In particular, longlasting synovial fluid induced a significant reduction of the severity of subsequent arthritis (p < 0.01) while SF from recent RA effusion (5–10 days after a previous complete extraction) and knee osteoarthrosis were ineffective. It is reasonable to assume there are at least two unknown endosomal immunoactive epitopes; one developing its immunotherapeutic property in RA, and the other, related to the molecule of HSP60, reduces the severity of oncoming arthritis. Both epitopes are present in humans, have a molecular weight of ≤10 kDa and do not appear to be bystander antigens. Please see Additional file 1 for the abstract in Italian.

**Electronic supplementary material:**

The online version of this article (doi:10.1186/s12967-016-0908-7) contains supplementary material, which is available to authorized users.

## Background

Rifamycin SV is an old antibiotic endowed with a cytolytic property that completely lyses synovial cells and most likely frees the autoantigen anchored on the binding groove of antigen presenting cells (APCs) [1, 2]. Rifamycin SV also provokes the release of all short proteins at various stages of enzymatic degradation from the endosomal compartment. The good therapeutic response to Rifamycin SV injected into the arthritic knee [3–7] and, in particular, the systemic effects obtained when the application is extended to many joints [8–10] have led us to hypothesize that this antibiotic, once infiltrated in joints, frees the pathogenic peptide from synovial APCs, which, in conjunction with MHC class II molecules, would otherwise present this peptide to autoreactive T cell receptors, contributing to the continued immune response. The peptide itself might then instead favor an interaction with the antigen-binding site of an anti-idiotype T-cell (paratope) [11, 12], activating an immunoregulatory response.

The idiotype-anti-idiotype network on membrane is considered central in immunoregulation involving autoantigens [13].

The use of a specific autoantigen at appropriate doses to bypass interaction with autoreactive T lymphocytes and thus detrimental immune response has been shown to cure spontaneous autoimmune disease, as with glutamic acid decarboxylate (GAD 65) in the non-obese diabetic rat [14]. Thus, the isolation and identification of autoantigens for other autoimmune disorders such as rheumatoid arthritis (RA) might have considerable therapeutic benefit. To facilitate this, the use of peripheral blood mononuclear cells (PMBCs) of subjects with RA as a source to extract the pathogenic peptides in vitro instead of the less accessible synovial cells has been considered.

This is based on the notion that, although very different in number, many peripheral blood cells including macrophages, dendritic cells, and B lymphocytes, possess functional characteristics that are commonly attributed to actual APCs [15, 16]. All potential APCs are capable of degrading the antigens in their endosomal compartment; the subsequent transfer of the resultant peptides to the cell surface and their presentation to lymphocytes gives rise to immunoinflammation [17].

Within PBMCs, research on the pathogenesis of systemic and organ-specific autoimmune diseases has expanded the complex role of B cells from the synthesis of antibodies and pro-inflammatory cytokines to that of internalization and presentation of antigens [18, 19].

B cells process and present the antigen peptides to CD4+ T lymphocytes in the context of MHC class II alleles. It has been argued that in established autoinflammatory conditions B cells represent a potent reservoir of APCs for activating T cells [20].

Starting from these considerations, we thought of inducing the release of pathogenic peptides from PBMCs in vitro by causing the lysis or fragmentation of cells through the use of Rifamycin SV as a cytolytic substance, or through freeze/thawing of cells, respectively. Separation of unknown PBMC substances in the tube could determine the leak of autoantigen as well as all the intracellular material in which there are many unknown substances.

The separation of peptides and any other substances from lysates and from the suspension of cell fragments could be obtained by centrifugation and subsequent immediate ultrafiltration (mw ≤10 kDa). All the molecules of low weight could be collected in the flow-through. Among these, in addition to the short proteins from the endosomal compartment, might be included the specific arthritogenic peptides.

The same method of separation could be applied to rheumatoid synovial fluid as well. In the context of synovitis, the release of pathogenic peptides is induced by inflammatory disruption of the synovial cells. The immediate ultrafiltration of centrifuged fluid would allow immunoactive peptides, free of molecules involved in the endosomal processing of antigens (e.g., class II-associated invariant peptide) and not linked to the molecule HLA DR, to be collected in the flow-through.

As first step in determining the identity of the autoantigen(s) in RA, in this study, we have prepared four ultrafiltrates, two of which were derived from the breakdown of PBMCs, one from the supernatant of PBMCs cultures and one from synovial fluid.

Three of these were utilized in trials of autologous immunotherapy by injecting them subcutaneously in patients with early RA (disease duration of less than 12 months) and for a protection protocol in adjuvant arthritis (AA) in rats (iv administration 7, 8 days after AA induction). We chose AA model because is considered a disease mediated by auto reactive T cells cross-reacting with mycobacterial hsp65 [21, 22], which is a stress protein highly conserved through the evolution, from bacteria to man, and yet the arthritogenic autoantigen(s) remains elusive [23].

## Methods

### Preparation of ultrafiltrates

All procedures were performed in BSL3. The ultrafiltrate preparation protocol was performed using sterile equipment in laminar flow cabinet with HEPA filter. The sterility of PBMC ultrafiltrates was tested by placing it in culture on Blood Agar and Muller Hinton broth.

#### Isolation of peripheral blood mononuclear cells

Peripheral blood mononuclear cells were isolated from whole blood using Histopaque-1077 polysucrose and sodium diatrizoate solution, according to manufacturer’s instructions

Briefly, whole blood samples from the patients were labeled with a univocal barcode defined by the protocol and diluted 1:2 with 1× PBS in a labeled 50-mL Falcon tube. In a second Falcon tube, 10 mL of Histopaque-1077 was added, onto which 20 mL of diluted blood was subsequently layered. Then, Falcon tubes were centrifuged at 750×*g* for 30 min at room temperature, without brake deceleration. The PBMCs were then collected, divided into four 15-mL Falcon tubes, and centrifuged three times at 650×*g* for 10 min, at room temperature, in 10 mL of 1× PBS solution to remove the red blood cells. Finally, PMBCs were counted and stored at −80 °C, at a final concentration of 5 × 10^6^ cells/mL, in tubes labeled with the appropriate protocol barcode.

#### Ultrafiltrate UF-S (mononuclear cell supernatant)

PBMCs (5 × 10^6^ cells/mL) were cultured in shell vials in RPMI 1640 medium containing 20 % fetal calf serum, at 37 °C, in a 5 % CO_2_-enriched atmosphere for 3 h. At the end of the incubation period, the supernatant was collected after centrifugation at 350×*g* for 10 min, ultrafiltrated by passage through a Millipore filtration device with a molecular weight cut-off value (MWCO) of 10,000 kDa (Millipore Corporation, Bedford, USA), and stored at −20° until use.

#### Ultrafiltrate UF-R

For the preparation of ultrafiltrate for use in immunotherapy, rifamycin SV, stored in 10-mL (250 mg) vials (Lepetit Spa, Milan) (for intravenous use), was utilized.

PBMCs (5 × 10^6^ cells/mL) were cultured under the same conditions, but in the presence of 250 μg/mL of rifamycin SV, for 3 h. At the end of the incubation period, the rate of cell lysis observed was 90 %. The supernatant was collected after centrifugation at 350×*g* for 10 min and the ultrafiltrate was prepared as described above. The flow-through was used within 2–3 days, and the remainder was stored at −20 °C. The ultrafiltrate prepared with rifamycin SV was used in clinical trials.

#### Ultra filtrates UF-f/t

PBMCs were purified using Histopaque-1077, as described above, but cell lysis was not performed.

Briefly, PBMCs were subjected to three freeze/thaw cycles from −80 °C to room temperature and centrifuged at 1500×*g* for 10 min; up to 1.5 mL of sample was then transferred into Millipore filtration devices with a MWCO of 10,000 kDa, and centrifuged at 1500×*g* for 10 min in labeled 50-mL Falcon tubes. The ultrafiltrate was collected in 2 mL tubes and stored at −20 °C.

It should be noted that epitopes bound to HLA-DR (>10 kDa) are too large to pass through the pores of the ultrafilter while the free pathogenic epitope and other endosomal peptide easily pass.

Generally, the ultrafiltrate was prepared a few days before its subcutaneous administration. A portion of the ultrafiltrate was stored separately for in vitro studies (see below).

#### Ultrafiltrate UF-sf

Synovial fluid from a long-lasting (for at least a month) inflammatory effusions, was centrifuged to remove the cells and then subjected to ultrafiltration using the procedures described above. The ultrafiltrate was used within 2 days, and the remainder was stored as above.

### Rheumatoid arthritis

#### Patients

Patients recruited for this study fulfilled the American College of Rheumatology (ACR) 1987 revised criteria for RA [24]; specifically, patients with disease duration of less than 1 year and who had not been previously treated with immunosuppressive agent were recruited. The inclusion criteria were as follow: age between 17 and 75 years, ACR functional class I, II or III [25], presence of active disease symptoms such as nine or more tender joints at rest or in motion, six or more swollen joints, a pain score of greater than four on the Visual Analog Scale (VAS) and an erythrosedimentation rate (ESR) of >30 mm/h. The exclusion criteria were as follow: known allergy to Rifamycin SV antibiotic, a positive intra-dermal sensitivity test for Rifamycin SV, the intra-articular or parenteral administration of corticosteroids 4 weeks prior to the study.

#### Study design and monitoring

In this study, three of four ultrafiltrate samples, which were prepared as described above, were used: UF-sf (prepared from rheumatoid acellular synovial fluid), UF-f/t (prepared by freeze/thawing of PBMCs), and UF-R (obtained by lysing mononuclear cells from patients using Rifamycin SV).

The objectives of this study were to investigate the efficacy of autologous ultrafiltrates, administered via subcutaneous injection at baseline on two consecutive days, and to compare the response rate on the 10th day vs those on the 30th day in each of the three treatment groups.

AS the groups were not homogeneous in terms of patient characteristics the response rates of the different groups to treatment could not be compared, therefore the groups were evaluated separately.

The choice of non-placebo group was based on ethical, practical and methodological considerations. Moreover, a single blind study design, in which the investigator evaluating the response was not aware of the treatment administered, ensured the absence of assessment bias.

The study design included randomization between groups; however after the first ten randomized patients 70 % of patients tested positive for intra-dermal sensitivity test to Rifamycin SV (wheal diameter ≥0.5 cm). This unexpectedly high percentage of patients sensitive to Rifamycin SV necessitated a change in the design of the study, in that the randomization procedure was no longer considered feasible.

The study design was modified according to the following criteria: patients (a) with a previous or current history of allergic or autoimmune diseases other than RA, such as atopic dermatitis, chronic hypersensitivity pneumonitis, leukopenia due to hypersensitivity, and asthma, (b) with clinical conditions that may be aggravated by Rifamycin SV such as elevation of liver enzymes, impaired creatinine clearance, cholelithiasis, (c) who may have been treated with the Rifamycin SV (e.g., patients with previous tubercular infection) and those who were treated with this antibiotic via the intrasynovial route for arthritis, and (d) nurses were excluded from the UF-R group and included in the UF-f/t group. In addition, only patients presenting synovial effusion of knees were included in the UF-sf group.

A total of 115 patients with active RA were enrolled in this study at the L. Sacco Hospital, 11 of whom had joint effusions of both knees. Patients in groups UF-R and UF-f/t were administered the previously prepared ultrafiltrate (1.7–2 mL) at subcutaneous sites on the forearm on two consecutive days, whereas those in the UF-sf group received 0.8–2 mL of ultrafiltrate upon availability. An adverse reaction to these antigens was unlikely as peripheral lymphocytes reactive to self peptides are eliminated during ontogeny via negative selection.

The study design was approved by the Ethics Committee of L. Sacco Hospital and by the Ethics Committee of the Regional Government of Lombardia, Italy (Resolution number 599 of July 30 2009). Informed consent was obtained from all patients. Patients underwent a complete physical assessment and 30–50 mL peripheral blood was drawn for blood tests and cell ultrafiltrate preparations and synovial fluid, where present, was completely drained. The following were evaluated for measurement of changes from the baseline at every assessment: swollen joint count, tender joint count, pain score (VAS), patient and physician global assessment of disease activity (VAS), and efficacy according to the ACR criteria [26]. Inspection of the injection site and general tolerability were evaluated at the 10th and 30th day of the clinical trial. An immune response to a subcutaneous administration of any antigen rarely lasted for more prolonged than 30 days. However, more detailed studies are necessary to define the schema of this immunotherapy.

### Rheumatoid arthritis—in vitro studies

#### Cells and ultrafiltrate

The cells for the cultures were obtained from a pool of about 50 mL whole blood, drawn from six patients with active rheumatoid disease. The cells, purified on Histopaque-1077 were frozen at 5–10 × 10^6^ cells/mL and stored in a cryogen tank until use. At the time of use, the PBMCs were thawed and centrifuged at 750*g* for 10 min, washed 3× with PBS, and resuspended at 0.5–2 × 10^6^ cells/mL DPBS (Dulbecco’s Phosphate-Buffered Saline. Carlo Erba. C Dasit group. Milan).

Cells were grown in RPMI1 1640 supplemented with 10 % heat inactivated human AB serum, 100 U/mL penicillin, 100 μg streptomycin, and 1 mM glutamine. The PBMCs were cultured at 0.5–2 × 10^6^ per well in a 48 well plate for 48 h with media alone, or stimulated with plastic bound anti-CD3 (clone OKT3) (5 μg/mL) and soluble anti-CD28 (2 μg/mL), 10 μg/mL tetanus toxoid-derived peptide (QYIKANSKFIGITE) or hsp60 (GEALSTLVVNKIRGT) (Becton–Dickinson). Cultures were set up in duplicate with one set receiving 400 μL (0.1 mg protein per well) of UF-sf. The PBMC ultrafiltrate was adjusted to 10 % human AB serum prior to addition to cell cultures. After 48 h, the cells were harvested and immunostained as described below. The UF-sf for the in vitro studies were the same as those prepared and employed as autologous vaccines.

#### Ultrafiltrates

For UF-sf, the samples stored at −20 °C were thawed and pooled in the laboratory for in vitro assays. A micro BCA protein assay (Pierce Biotecnology, Rockford, IL) was performed to determine the protein concentration of the pooled ultrafiltrates. The ultrafiltrate prepared with Rifamycin SV was excluded from this study because it cannot be removed from the extract, making in vitro studies difficult.

#### SDS-PAGE and western blot analysis

Several experiments were performed to attempt to visualize the protein of interest on a gel. These experiments were performed with some of the patient samples as well as with samples from donors recruited for this purpose. The ultrafiltrates prepared with Rifamycin SV were run on 4–12 % SDS-PAGE gels and silver staining and western blot analysis with anti-hsp60 (Becton–Dickinson, San Diego, CA), as the detection antibody, were used for protein visualization.

#### FACS analysis

Cell staining was used to measure (1) TNF-alpha production by T cells and macrophages, (2) CD69 as an indicator of cell proliferation and (3) CTLA-4 expression by CD4+/CD25+ cells as an inhibitor of T cell function. Isotype controls were included to determine non-specific antibody staining. Monensin was added to the PBMC cultures 4 h prior to immunostaining, and the cells were harvested and extracellular immunostaining was performed with antibodies to human CD3 (clone UCHT1), CD4, CD25, CD69 and CD11 (Becton–Dickinson). The cells were fixed with paraformaldehyde and intracellular staining was performed with antibodies to TNF-alpha and CTLA-4 in the presence of saponin. Appropriate isotype controls were also included.

#### Ultrafiltrate from a normal donor is not toxic to normal BPMCs

In order to test the possible toxicity of ultrafiltrate on cells and to determine the appropriate volume to use for subsequent patient studies, the effects of normal donor ultrafiltrate on CD69 expression, a marker for cell proliferation, by T cells in cultures with normal PBMCs, were evaluated. PBMCs were cultured with increasing volumes of UF-f/t (from 0 to 500 μL) in the presence or absence of anti-human CD3 (5 μg/mL) and CD28 (2 µmL). Cells were incubated for 24 or 48 h and co-stained with anti-CD3 and anti-human CD69. The proliferation of stimulated cells, as measured by the expression of CD69, (% CD69/CD3), was consistently high with all volumes of ultrafiltrate.

Not being toxic the maximum feasible amount of ultrafiltrates (400 µ) were used. Additionally, CD69 expression was higher at 48 h and that cultures time was used (the tables are available on request).

### Adjuvant arthritis in rats

#### Animals studies were performed at the LITA laboratories, Microbiology Chair, University of Milano (Italy)

##### Induction and assessment of arthritis

Adjuvant arthritis (AA) was induced by intracutaneous injection of 0.1 mL of paraffin oil containing 10 mg/mL of heat-killed *Mycobacterium butirricum,* into the base of the tail of female Lewis rats. Active disease developed 11–12 days after injection reaching a peak at 17–21 days; this was followed by slow resolution. Severity of arthritis was evaluated every fourth day by the measurement of clinical signs in each paw, on a scale of 0–2. The maximum arthritis score was eight per animal. The rats lost 5 % of their body weight over the course of the clinical disease.

##### Protection protocol using ultrafiltrates

Fifty rats were randomized into the following five treatment groups: Rifamycin SV alone (250 μg/mL), saline solution, UF-S, UF-f/t or UF-R, and intravenously treated, with 0.5 mL/rat/day, on days 7 and 8 after AA induction. We did not administer ultrafiltrate during the overt manifestation of arthritis.

##### Collection of synovial fluid

Synovial fluids were obtained from eight patients with rheumatoid arthritis with long-lasting knee effusions, six with recent effusions (5–10 days after a previous complete extraction) and six with arthrosis. Synovial fluids were centrifugated at 400×*g* for 15 min; the supernatant was the ultrafiltrated as above and stored at −20 °C until use.

##### Protection protocol using ultra filtrate UF-sf

Animals (n = 8) were injected in the tail vein with 0.5 mL of UF-sf or saline solution (control) (8). Each animal was treated with UF-sf obtained from an individual patient. Twenty-one days after AA induction the arthritis severity was scored by grading each hind paw.

### Statistical analysis

#### In vitro studies

Changes from the 10th to the 30th day were assessed using a Chi square test for the UF-f/t and UF-R groups using 95 % confidence intervals, whereas the Fisher’s exact test was used for the third comparison with the UF-sf group, because of the low number of patients therein. For immunohistochemistry, nonspecific staining as determined with the isotype controls was subtracted (as percentage) from the positive responses. Differences between matched untreated PBMCs and samples treated with ultrafiltrates were analyzed by the Wilcoxon paired rank test.

#### Clinical studies

All data concerning the protective protocol in rats were analysed with non-parametric tests. To compare the different groups, the Friedman’s test was performed with time and treatment as variables. Synovial fluid results were analysed by one way analysis of variance followed by Bonferroni T test for multiple comparisons.

## Results and discussion

### Rheumatoid arthritis

The results reported in Table 1 show that the three autologous ultrafiltrates, administered subcutaneously, led to good therapeutic effects. In some patients, these effects occurred early, sometimes after just a week, and lasted at least until the final check of the 30th day. In the UF-R group, 37 % (17/46) of the patients achieved an ACR20 by the 10th day vs 33 % (19/58) of those receiving UF-f/t. The patients who received UF-sf showed a still higher ACR20 response rate of 64 % (7/11). In the subsequent 20 days, the number of patients with a positive therapeutic response increased slightly in UF-R and UF-f/t, from 17 to 19 (41 %) and 19 to 22 (38 %), respectively, and the final results were comparable between these groups. In contrast, patients in the UF-sf group remained unchanged (64 %). The comparison of the results between the 10th and the 30th day did not show any significant difference in any of the three groups.

**Table 1:** Disease activity score after subcutaneous injection of autologous ultrafiltrates in early rheumatoid patients

Ultrafiltrates^a^	Pts	ACR ≥ 20^b^ (10th day) vs (30th day)		RR°	95 %	CI	p value
		(%pts10th day)	(%pts 30th day)				
UF-R rifamycin SV (from 5 × 10^6^ cells/mL)	46	17 (37)	19 (41)	0.90	0.54	1.49	NS
Ul-f/t freeze/taw (from 5 × 10^6^ cells/mL)	58	19 (33)	22 (38)	0.86	0.53	1.42	NS
UF-sf (from acellular sin. fluid)	11	7 (64)	7 (64)	1	0.53	1.88	–

^a^One subcutaneous administration in two consecutive days. For doses, see text

^b^American College Rheumatology criteria ≥20° relative risks (RR), calculated using Chi square Pearson test

Among the three ultrafiltrates, UF-f/t would appear to be preferred for clinical use although its response rate by the 30th day was the lowest (22/58, 38 %), because it is the easiest to prepare and can be prepared and administered the same morning. On the other hand, UF-R has the disadvantage of potential patient hypersensitivity reaction to the antibiotic, Rifamycin SV, which cannot be eliminated from the PBMC lysates (see safety). It has been reported that serious side effects occur in patients who take the Rifamycin SV intermittently [27]. Finally, UF-sf presents the greatest obstacle for practical use because only a minority of patients have abundant synovial fluid in their knees.

### Rheumatoid arthritis in vitro studies

As previously suggested, we expect that the immune-active epitope should be the same between the three ultrafiltrates. Therefore, for the in vitro studies, we employed the ultrafiltrates UF-f/t and UF-sf but not UF-R, as it was prepared with Rifamycin SV.

Notably, in the latter the Rifamycin SV itself was the only low molecular weight protein detected (SDS-PAGE), which might have interfered with the detection of other small molecular weight proteins. The Western blots also showed no proteins when an anti-heat shock protein antibody was used for detection.

### Immunomodulatory activity of ultrafiltrates on PBMCs from patients with RA

For this study we chose to test the ultrafiltrate UF-sf because the concentration of the pathogenic epitope in synovial fluid could be greater than in the ultrafiltrate UF-f/t.

This we have deduced from experiment reported in Table 4 (see below) in which longlasting synovial fluid was significantly more effective than the recent one in the inhibitory activity against AA showing the ability of the pathogenic epitope to accumulate.

**Fig. 1:**
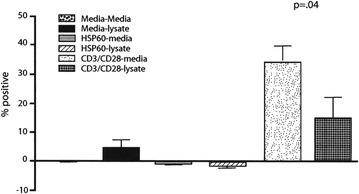
Expression of CD69 by CD4+CD25+ cells from six RA patients. Results are shown as the mean and standard deviation of the six patients. Only anti-CD3 and CD28 stimulate the cells to proliferate and this proliferation as measured by CD69 was significantly decreased by the addition of lysate

**Fig. 2:**
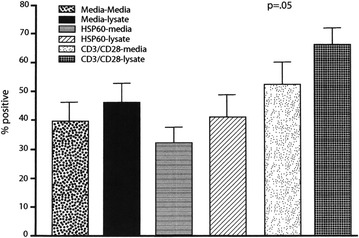
Expression of CTLA-4 by CD4+CD25+ cells from six RA patients. Results are shown as the mean and standard deviation of six patients. CTLA-4 by the same cell population increase slightly despite the decrease in CD69 expression (not shown) and was significant in the case of the CD4+CD25+ T cells

PBMCs (1 × 10^6^ per well) patients with active RA (6) were cultured in duplicate with UF-sf and stimulated with anti-CD3/CD28. After 48 h, the cells were harvested and immunostained for CD4/CD25, CTLA-4, CD69, CD11c and TNF-alpha. Very little TNF-alpha expression resulting from any of the treatments (peptides) was identified. Only anti-CD3 and CD28 stimulated the cells to proliferate and this proliferation as measured by CD69 was significantly decreased by the addition of ultrafiltrate (p = 0.04) (Fig. 1). CTLA-4 expression (a marker of T regulatory cells) by the same cell population increased slightly (not shown) despite the decrease in CD69 expression and was significant in the case of CD4+CD25+ T cells (p = 0.05) (Fig. 2). For background correction, nonspecific staining as determined using the isotype controls, was subtracted from the positive responses.

### Immunological response to autologous PBMC ultrafiltrates in patients with RA previously vaccinated (30 days) with autologous UF-f/t

PBMCs from one normal subject and three patients with early RA who had been previously vaccinated, i.e., subcutaneously treated with autologous UF-f/t, were cultured with the same autologous ultrafiltrate and then immunostained. When the Wilcoxon paired samples test was used to determine the differences between no treatment and subcutaneously treatment with autologous ultra filtrates, no statistically significant differences were observed for any of the markers studied (Table 2). However, when CTLA-4 expression by CD4+CD25+ T-cells was examined, the addition of ultrafiltrate decreased the level of this marker in normal donors (−33 %), which appeared to correlate with the decrease in CD69+ expression. The opposite effect was observed in the autologous vaccinated patients with RA, who exhibited a decrease in CD69+ by CD4+CD25+ T cells but no change in CTLA-4 expression by the same cells following the addition of ultrafiltrate. In addition, there was little TNF-alpha production by either T cells or monocytes/macrophages.

**Table 2:** The effects of autologous ultrafiltrate on CD69 and CTLA-4 expression by PBMCs stimulated with CD3/CD28

Donor	% CD69+/CD4+	% CD69+/CD4+CD25+	% CTLA-4+/CD4+	% CTLA-4+/CD4+CD25+
Normal (n = 1)	−25	−18	+4	−33
Patients (n = 3)	−7	−15	+4	+3

The results are shown as the untreated cells minus cells plus lysate. CD69 and CTLA-4 were down-regulated in the normal donor

### Synthesis

- The addition of UF-sf to PBMCs cultured from non-vaccinated patients with RA (6) elicited significant reduction of CD69 by CD4+ cells (p = 0.04) and at the same time an increase of CTLA-4 expression by CD4+CD25+ cells (p = 0.05).
- The addition of autologous UF-f/t to PBMCs cultured from vaccinated patients elicited minimal change in cellular markers.
- The addition of autologous UF-f/t decreased the expression of both CD69 (−25 %) and CTLA-4 (−33 %) in normal subjects.
- The addition of antigen p1 (HSP65) did not show any response.
- Addition of the ultrafiltrate did not elicit a pro inflammatory response in any of the studied subjects: normal donor, patients with RA, or patients with RA previously vaccinated with autologous PBMC ultrafiltrate.

### Adjuvant arthritis

Table 3 reports the clinical scores of AA. Prevention of paw swelling after treatment with UF-S and UF-f/t was statistically significant (p < 0.01) compared with the control groups. These results showed that active unknown epitopes would be released in UF-S even during 3 h of culture (in the absence of Rifamycin), following a partial spontaneous cytolysis. On the contrary, AA was not inhibited in rats treated with UF-R and Rifamycin alone (Table 3) and also by the UF-sf from patients with arthrosis (Table 4). Arthrosis is commonly accepted as the normal control for RA.

**Table 3:** Modulation of adjuvant arthritis development in rats by administration of ultrafiltrates obtained from PBMCs of RA patients

Days from AI	UF-S**		UF-f/t**		UF-R		Rifamycin		Saline sol.	
	AA score	Median	AA score	Median	AA score	Median	AA score	Median	AA score	Median
11	00000 00233	0	00000 11246	0.5	00122 22223	2	00001 11222	1	00011 22245	1.5
15	00012 24457	2	02222 33357	2.5	12223 33344	3	02333 33444	3	24445 55666	3
19	11244 45588	4	02344 45557	4	24555 66788	5.5	25555 66788	5.5	46666 67777	5.5
23	13333 55788	4	03344 55567	4.5	24555 66888	5.5	33344 56668	4.5	45555 66678	4.5
27	12223 36778	3	02344 44457	4	23455 55888	5	33334 55668	4.5	44445 55668	4.5

Ten rats per group were injected with 0.5 µg/mL of each ultrafiltrate or 0.5 mL of Rifamycin SV (250 µmL) or saline solution at 7 and 8 days after arthritis induction

*AA* adjuvant arthritis, *AI* arthritis induction, *RA* rheumatoid arthritis

** p < 0.05 vs saline solution

**Table 4:** Protection of rats from adjuvant arthritis by administration of synovial fluid (UF-sf)

Longlasting RA synovial fluid (N = 8)	Recent RA synovial fluid 5–10 days (N = 6)^a^	OA synovial fluid (N = 6)	Saline solution (N = 8)
2.37 ± 1.60º	5.9 ± 1.01^	6.7 ± 095^	7.5 ± 0.92^

Clinical score at 21 days after arthritis induction

*RA* rheumatoid arthritis, *OA* arthrosis

º p < 0.01 vs saline solution

^a^After a previous complete extraction

^Not significant. Each rat was treated with synovial fluid of an individual patient

The administration of long-lasting (more than a month) RA synovial fluid, UF-sf, induced a significant reduction of the severity of subsequent arthritis in rats (p < 0.01) while SF from recent RA effusion (5–10 days after a previous complete extraction) was ineffective (Table 4). The ineffectiveness of the recent fluid is probably due to the gradual accumulation over time of immunoactive epitopes from lysis of proliferating synoviocites and deeper mononuclear cells in inflamed synovium. The control saline treated animals had a severe arthritis, as demonstrated by the high clinical scores (Table 4).

In conclusion, longstanding synovial fluid, UF-sf, the supernatant of PBMC cultures, UF-S, and Ultrafiltrate of PBMCs fragmented with freeze/thawing, UF-f/t, all from subjects with RA, are able to modulate the immune mechanism implicated in AA and to stimulate protective immunity.

In Table 5, we summarize the results of autologous immunotherapy in RA patients and the protection profile of the human ultrafiltrates administered in rats with oncoming arthritis.

**Table 5:** Autologous immunotherapy of RA with ultrafiltrates derived from PBMCs and synovial fluids. The protection profile in AA is included

Lysate ultrafiltrates	Abbreviation	RA patients autologously injected	Adjuvant arthritis (AA) in rats iv injected
Prepared from acellular synovial fluid	UF-sf	Active	Active
Prepared by freeze/thawing of PBMCs	UF-f/t	Active	Active
Prepared from supernatant of PBMCs culture	UF-S	ND	Active
Prepared by lysing PBMCs with rifamycin SV	UF-R	Active	Ineffective

*RA* rheumatoid arthritis, *AA* adjuvant arthritis in rats, *ND* not done

Our strategy to extract and identify pathogenic epitopes was linked to the notion that in a systemic disease such as RA, the epitope would be present in the APCs of PBMCs as well as in synovial cells. Recently, the presence of pathogenic-like T cells has been demonstrated in the blood stream of adult forms of autoimmune arthritis and juvenile chronic arthritis; these T cells share genotypic and phenotypic characteristics with synovial T cells [28]. Thus, the presence of pathogenic lymphocytes implies the presence of APCs as well.

The ultrafiltration of lysate and/or the suspension of cellular fragments immediately after centrifugation were intended to retain the proteolytic enzymes and allow numerous unknown small molecules, including the possible autoantigen, to pass into the ultrafiltrate aqueous solution that was used for subcutaneous injection. This extremely simplistic theory is supported by the development of an indisputable efficacy of the ultrafiltrates in patients with arthritis. Most notably, the therapeutic effect lasting 30 days from a subcutaneous dose is a type of response that differs completely from all known anti-arthritic therapies [29].

In comparison, the administration of a specific, known autoantigen in appropriate doses, such as glutamic acid decarboxylate (GAD 65) in the non-obese diabetic rat, a well-known animal model of spontaneous autoimmune disease, has been shown to cure the disease [14].

In the present study, the two immunotherapeutic vaccines prepared from the rheumatoid PBMCs, UF-f/t and UF-R, reached different outcomes in the two diseases; the UF-R was unable to modulate the animal model of arthritis but was effective in patients with RA while the UF-f/t worked both in AA of rats, reducing the severity of oncoming arthritis, and in patients with RA leading to an improvement of arthritic symptoms (Tables 1, 5).

The other two ultrafiltrates, UF-S and UF-sf, exhibited immunotherapeutic activities comparable to that of the ultrafiltrate prepared by freeze/thawing of PBMCs (Table 5).

It is reasonable to assume that there are at least two unknown immunoactive epitopes, in both mononuclear cells and synovial fluid ultrafiltrates.

These epitopes are thrown out of disrupted cells, specially from their endosomal compartment, during the preparation of ultrafiltrates. They are not joined to any HLA DR molecule, they pass through an ultrafilter with a cut off of 10 kDa (see “Methods” section) and are free of molecules involved in the endocytic processing of antigens (e.g., class II-associated invariant peptide).

The Rifamycin SV might have tied the epitope effective in AA, while preparing ultrafiltrate UF-R, but leaving intact the epitope effective in human arthritis. This results in the disabling of its property to protect rats from experimental arthritis.

The epitopes (two or more) of synovial fluid, spontaneously released following the lysis of proliferating synoviocites and deeper mononuclear cell of inflamed synovium, accumulate over time. If drawn out again, just a few days after a complete extraction of the effusion, this rheumatoid synovial fluid was ineffective on prevention of AA, unlike longlasting synovial fluid (Tables 4, 5). This might explain the previous reports on depressed proliferative response by PBMCs to mHsp60 in early RA [30]. The synovial fluids from arthrosis patients with arthrosis were always ineffective in modulating the AA of rats because they do not contain arthritogenic epitopes.

We know very little about the epitope effective in RA patients. It is present in both ultra filtrates of PBMCs and synovial fluid; it is effective as immunotherapeutic approach and exhibits immunomodulatory activities when is added to rheumatoid cell cultures (see below). We do not know the amino acid structure and the precise molecular weight. But It is just as small, much smaller than those identified with the p*an*-*DR*-*binding Hsp*-*derived epitopes* [31], as candidate for antigen specific bystander immunotherapy [32–35].

Once subcutaneously injected in RA patients, the epitope is transported by dendritic cells in inflammatory sites.

The status of the auto antigen in the ultrafiltrate, i.e., a peptidic fragment, not associated with a class II MHC molecule, might assist in explaining the early appearance of signs and symptoms of clinical improvement: being taken up and transported by dendritic cells, the self-epitope would likely interact directly with the antigen binding site of anti-idiotypic T cells on the cellular membrane (paratope), thus activating an immunoregulatory response. The idiotype-anti-idiotype network on the cell membrane is considered central in immunoregulation involving auto antigens. Therefore, the peptide would not interact with autoreactive T lymphocytes and thus would bypass the most detrimental component of the immune response in patients with arthritis. In fact, pro-inflammatory responses have never been observed in vivo or in vitro.

We hypothesized that the intrasynovial Rifamycin SV [1, 2], through these autonomic immunological mechanisms established an endogenous immunotherapy.

The PBMCs from RA patients, in baseline cultures, were unresponsive to synthetic HSP60 bystander peptide P1 (Fig. 1) but exhibited an immune response to the addition of the ultra filtrate UF-sf which was characterized by significant increase in the expression of CTLA-4 by CD4+CD25+ (p = 0.05) and an equally significant reduction of CD69 (p = 0.04); modifications which jointly imply an immunoregulatory response that only a specific antigen is able to elicit in subjects that have, in all probability, a basic immunological trend potentially oriented toward immunoregulatory responses.

In this context, a certain role might be played by the innate immunity that is persistently activated in patients with RA [36, 37].

The addition of autologous UF-f/t to PBMCs cultured from vaccinated patients elicited minimal change in cellular markers (Table 2). This might indicate that mononuclear cells are functionally impaired by previous in vivo stimulation. In contrast, in cultures from normal subjects there was a marked reduction in the expression of CTLA-4 (−33 %) after the addition of autologous ultrafiltrate (Table 2). This finding reinforces the speculation that subjects with RA have a particular arthritic immunological profile, particularly earliness of clinical improvement and the duration of a single subcutaneous injection.

As regards the immune response in rats with ongoing arthritis, the mechanisms of protection might be the same with the difference that, being induced, the disease is self-limiting.

However, the epitope injected with human ultra filtrate, very likely related to HSP60 molecule, remains unknown. As the HSP is an extraordinarily evolutionarily conserved molecule the presence of its epitopes in the endocytic compartment of human cells is almost obviously. It is well known that T cells specific for self-Hsp even exist in the normal T cell repertoire.

Immune response to hsp60 by rheumatoid synovial cells and the presence of these antigens in inflamed joints and subcutaneous nodules of RA patients were reported [38, 39]. In JCA this responsiveness was directed to self hsp60 and the increased response of Hsp60-specific T-cells correlated with the spontaneous disease remission [40].

Notably, the epitopes contained in our ultrafiltrates and responsible for the prevention of AA, appear to be smaller than the aa sequence 180–188 of the mycobacterial HSP65 which, while preventing the AA and despite having therapeutic effects on this experimental model, is not considered as the pathogenic autoantigen (it is not able to induce the disease) [41].

The auto antigens extracted here, can be enriched many times (w/v), along with all the other small molecules, by aggregating multiple ultrafiltrates and subjecting them to lyophilization. Thus prepared, the ultrafiltrate might also be autologously used in non-rheumatological autoimmune disease, particularly in cases where the number of APCs in the blood is low, even if the autoantigen is unknown (personal obs). An adverse reaction to these antigens is unlikely given that peripheral lymphocytes reactive to self peptides are eliminated in the ontogenic selection.

In summary, this study outlines the unusual properties of the unknown and very probably pathogenic peptide that is contained in the ultrafiltrates prepared from rheumatoid PBMCs and synovial fluid. However, at the same time, the positive responses following subcutaneous administration of ultrafiltrates highlights the immunological profile of patients with RA, characterized by the potential capacity to reactivate, as a result of a specific stimulus, a pre-existing protective mechanism that is, of itself, insufficient to produce clinically significant effects.

The same human ultra filtrates contain an epitope, related to the molecule HSP60, that is able to modulate the immune mechanism implicated in AA and to stimulate protective immunity.

These studies should be considered very preliminary, and the clinical data must be interpreted with caution in view of the small number of cases.

No side effects were observed in our study following patient treatments, including at the site of subcutaneous administration of UF-sf or UF-f/t. However, on occasion the beneficial response was preceded by slight exacerbation of symptoms. Two patients in group UF-R experienced a hypersensitivity reaction after the second injection; both were characterized by skin eruptions that regressed spontaneously in a few days. In 13 patients, the intradermal sensitivity test to Rifamycin SV prior to enrollment was considered positive (appearance of wheal ≥0.5 cm). None of our patients experienced side effects common to immunotherapies in humans and in most studied experimental models of autoimmune diseases such as diabetes and multiple sclerosis (hypersensitivity reactions, immunization towards cryptic epitopes, or de novo induction of autoimmune diseases).

## Conclusions

- The disruption of PBMCs by the lytic action of Rifamycin or by freeze/thawing in vitro, caused the release of endosomal content and pathogenic autoantigens from APCs present in the circulation, which were then be isolated from degrading enzymes and bound immunologic molecules by ultrafiltration.
- The method of recovery, which utilize an ultrafilter with a cut-off of 10 kDa, lead to the conclusion that the possible pathogenic peptide is not associated with HLA-DR molecules.
- We prepared four different ultrafiltrates, two of which were derived from the breakdown of PBMCs, one from the supernatant of PBMCs cultures and one from synovial fluid. Three of these were utilized in trials of autologous immunotherapy by injecting them subcutaneously in 115 patients with early RA (disease duration of less than 12 months).
- At clinical monitoring on the 30th day, 22/58 (38 %) patients treated with the autologous ultrafiltrate prepared by the freeze/thawing of PBMCs reached an ACR20. But in some patients these effects occurred early, sometimes after a week.
- The status of the auto antigen in the ultrafiltrate, i.e., a peptidic fragment, not associated with a class II MHC molecule, likely taken up and transported by dendritic cells, allows the administered pathogenic peptide to by-pass an interaction with autoreactive T cells and favor interaction with the anti-idiotype (paratope) antigen binding sites, activating an immunoregulatory response. The early appearance of clinical improvement in the patients might be related to this mechanism of response.
- Examining the results obtained in rats with AA and in subjects with RA it is reasonable to assume there are at least two unknown immunoactive epitopes: one, derived from the molecule of HSP60 (HSP is highly evolutionarily conserved molecule), which causes immunosuppression in rats, the other develops its immunotherapeutic capacity in RA patients. They are both present in humans.
- In vitro studies defined certain notable immunomodulatory properties of the ultrafiltrates, and thus of the peptide, and outlined a particular immunological profile of the subjects with RA.
- During the course of the immunotherapy with freeze/thaw and acellular synovial fluid ultra filtrates, no side effects of any type were observed, nor were hypersensitivity or anaphylactic reactions induced. Adjuvant of any sort was never used. However, two subjects treated with ultrafiltrate prepared with Rifamycin SV experienced a morbilliform rash.

## Perspectives

In agreement with the preliminary results and considering the huge amount of knowledge in the field of human immunotherapy and in the prevention of experimental autoimmune diseases, we suggest that the use of ultrafiltrates, with the appropriate adjustment of the scheme, be applied for a perspective clinical study aimed at preventing the appearance of RA in subjects with a genetic profile associated with elevated risk for RA and/or a high level of anti-citrullinated protein antibodies. Furthermore, as autoantigen(s) can be enriched many times (w/v) through aggregation of multiple ultrafiltrates followed by lyophilization (Caruso unpublished), this method might also be applied in non-rheumatological autoimmune disease, particularly where the number of APCs in the blood is low

## Additional file

10.1186/s12967-016-0908-7 RIASSUNTO.

## Abbreviations

UF-R: ultrafiltrate obtained by lysis of PBMCs with Rifamycin SV
UF-sf: ultrafiltrate obtained from acellular synovial fluid
UF-f/t: ultrafiltrate obtained by freeze/thaw of PBMCs
UF-S: ultrafiltrate obtained from supernatant of PBMC cultures
