# Supplementary material for: Vaccination with endosomal unknown epitopes produces therapeutic response in rheumatoid arthritis patients and modulates adjuvant arthritis of rats
Source: J Transl Med. 2016 Jun 7;14:162. doi: 10.1186/s12967-016-0908-7 (PMC4897890; doi:10.1186/s12967-016-0908-7)
Supplement: Supplementary file 1 — 10.1186/s12967-016-0908-7 RIASSUNTO. [file 12967_2016_908_MOESM1_ESM.docx]

**Additional file 1**

**RIASSUNTO**

**INTRODUZIONE**Nostri studi precedenti hanno dimostrato che la rifamycina SV intrarticolare causa la lisi delle APC sinoviali e mette in libertà gli antigeni endosomali, non legati ancora a nessuna molecola HLA-DR. Questi stimolano una risposta immunoregolatrice instaurando clinicamente una immunoterapia endogena.

Qui, noi abbiamo ipotizzato che il disfacimento dei mononucleati periferici, col congelamento/scongelamento oppure attraverso l’azione litica della Rifamicina SV in coltura, causa la dismissione del contenuto endosomale e degli autoantigeni dalle APC circolanti i quali possono essere sottratti all’azione degli enzimi proteolitici mediante l’ultrafiltrazione.

**METODI**

Ii metodo di preparazione degli ultrafiltrati si basa sulla disgregazione delle PBMCs (5x10^6^ cells/mL) mediante l’aggiunta di Rifamicina SV in coltura (250µg/mL), la quale provoca la lisi del 90% delle cellule in 3 ore, oppure mediante 3 cicli di congelamento/scongelamento delle PBMC da-80° C a temperatura ambiente. Il lisato e la sospensione di cellule frammentate sono centrifugati e ultrafiltrati (cut off di 10kDa). Anche il liquido sinoviale acellulare è di lunga durata viene sottoposto a ultrafiltrazione.

**RISULTATI E CONCLUSIONI**

Al controllo del 30° giorno, il 38% (22/54) dei pazienti trattati per via sottocutanea con l’ultrafiltrato autologo preparato col congelamento/scongelamento delle PBMC ha raggiunto lo score ACR20.
Lo studio *in vitro* ha messo in evidenza che l’ultrafiltrato, aggiunto alle colture di mononucleati reumatoidi, induce una aumentata espressione di markers della immunoregolazione e una riduzione di quelli della proliferazione cellulare; modificazioni che congiuntamente hanno il significato di una risposta globale immunomodulante che soltanto un antigene specifico (ultrafiltrato UF-f/t) potrebbe indurre nel paziente reumatoide, verosimilmente attivando meccanismi protettivi preesistenti.
Artrite da adjuvante. Tutti gli ultrafiltrati, tranne quello preparato con la Rifamycina SV, hanno prodotto una modulazione significativa della susseguente artrite sperimentale nel ratto. Il liquido sinoviale longstanding, a differenza di quello recente e di quello artrosico, provoca la prevenzione della susseguente artrite (p<0,01).
Dal confronto dei risultati ottenuti nella RA e nella AA del ratto si desume che ci sono almeno due epitopi immunoattivi endosomali; uno, sviluppa le sue proprietà immunoterapeutiche nell’uomo e l’altro, derivato dalla molecola della HSP60, riduce in modo significativo la severità della susseguente artrite del ratto. Entrambi gli epitopi sono presenti nell’uomo, hanno un peso molecolare ≤ 10kDa e non appartengono agli antigeni comunemente detti bystander.
